# Current State of Point-of-care Ultrasound Usage in Canadian Emergency Departments

**DOI:** 10.7759/cureus.4246

**Published:** 2019-03-13

**Authors:** Mason Leschyna, Erfun Hatam, Samantha Britton, Frank Myslik, Drew Thompson, Robert Sedran, Kristine VanAarsen, Sarah Detombe

**Affiliations:** 1 Family Medicine, London Health Sciences Centre, University of Western Ontario, London, CAN; 2 Emergency Medicine, London Health Sciences Centre, University of Western Ontario, London, CAN; 3 Family Medicine, Queen's University, Kingston, CAN; 4 Emergency Medicine, Schulich School of Medicine and Dentistry, University of Western Ontario, London, CAN

**Keywords:** emergency medicine, point of care ultrasound, pocus

## Abstract

Background

Point-of-care ultrasound (POCUS) has many applications in emergency medicine, which have been proven to improve patient outcomes. Training programs and well-established guidelines for its use are available, but Canadian adoption rates and attitudes toward this technology have not been recently assessed.

Objectives

This study aimed to provide a national assessment of the current use of POCUS in Canadian emergency departments (ED) including patterns of use, attitudes towards its role, descriptors of training experience, as well as barriers to increased utilization.

Methods

An electronic survey was sent to physician members of the Canadian Association of Emergency Physicians. The survey included questions related to demographics, attitudes towards POCUS, POCUS utilization, and barriers to POCUS use. Responses were statistically analyzed to identify significant associations.

Results

Responses demonstrated a strong association between POCUS training and amount of POCUS usage. Neither hospital type nor community type was associated with the degree of POCUS usage. POCUS was most widely adopted for Canadian Point of Care Ultrasound Society (CPOCUS) core applications and has increased since the last national survey. The most commonly reported barrier to increased POCUS adoption was the lack of training. Most physicians have formal POCUS training in core applications, and approximately one third have advanced training.

Conclusions

POCUS training and utilization appear to have increased since the last national assessment. This provides a foundation for future POCUS research.

## Introduction

Adoption of ultrasound in the emergency department (ED) was first reported in 1988 [1]. Since then, advances in this technology have resulted in the rapid adoption of point-of-care ultrasound (POCUS) in emergency care with many applications well established by guidelines [2-3]. POCUS usage in the ED has been described as improving patient satisfaction as well as the physician-patient relationship [4]. There is support in the literature regarding the use of POCUS for many ED applications, including but not limited to: focused assessment with sonography in trauma (FAST), focused echocardiography, assessment of early pregnancy, musculoskeletal injuries and procedural guidance [5-9]. Multiple studies have found that after appropriate training physicians are able to successfully incorporate this tool into various aspects of practice [10-11]. POCUS usage in the ED also increases physician confidence with regard to the diagnosis and treatment decisions [12].

By comparing recent studies in the United States done by Moore et al. and Sanders et al., it is estimated that POCUS availability in US community EDs increased from 19% to 33% to 52% over a six-year period. Further, they demonstrate that, at that point in time, POCUS was most commonly used for focused assessment with sonography in trauma, assessment of the abdominal aorta and procedural guidance [13-14].

Despite the clear benefits of POCUS usage and rapid expansion of its utility in the ED, Canadian adoption rates and attitudes toward this technology have not been recently assessed. A Canadian census of past, current, and projected POCUS usage, as well as barriers to adoption, was most recently performed by Woo et al. in 2007 using the Canadian Association of Emergency Physicians (CAEP) survey distribution database [15]. They found the number of survey respondents who reported “always” using POCUS for the following Canadian Point of Care Ultrasound Society (CPOCUS) core applications was FAST 41.8%, basic cardiac assessment 30.0%, early pregnancy assessment 23.3%, and abdominal aortic aneurysm assessment (AAA) 30.7%. CPOCUS defines core POCUS applications as representing “ a highly limited use of ultrasound in potentially life-threatening situations when there is no time to involve radiological colleagues” [16]. The percentage of respondents that reported they would use POCUS for those same applications in the future was: FAST 88.4%, basic cardiac assessment 87.5%, early pregnancy assessment 73.7%, and AAA 92.6%. Since 2007, some regional Canadian studies have been conducted, specifically in rural EDs [16-17]; however, these respondents do not reflect the demographics of the entire country.

In 2008, POCUS became a core competency in the Fellow of the Royal College of Physicians of Canada (FRCPC) emergency medicine program, and in 2010, POCUS was declared a terminal training objective in the Certification in the College of Family Physicians Emergency Medicine (CCFP-EM) program [18]. As of 2012, POCUS has been incorporated into 100% of FRCPC-EM programs and 88.0% of CCFP-EM programs [19]. In 2018, all FRCPC EM programs transitioned to a competency-based residency curriculum [20]. Performing and interpreting POCUS to guide patient management is one of the entrustable professional activities (EPAs) in which competence must be achieved before completion of the program. CCFP-EM programs across Canada will also transition to a competency-based curriculum in the near future and will undoubtedly include POCUS in their list of EPAs as well. 

This study aims to provide a Canadian national assessment of the current state of EM POCUS, including demographics attitudes toward its role in the ED, POCUS applications and utilization, as well as barriers to utilization. 

## Materials and methods

Study design and target population

A link to an anonymous online survey in English was distributed via email to all staff adult emergency physician members of the CAEP survey distribution list in December 2016. The CAEP database was used because it is the largest group of its kind in Canada with members in every province and territory as well as all different types of EM training. Pediatric emergency physicians, residents, and medical students who were members of CAEP were excluded. Two weeks after the initial email, a reminder letter was sent to prompt non-responders.

Survey development

The survey was developed after a thorough search of the literature regarding emergency medicine applications of POCUS. Notable resources included the 2012 CAEP position statement on POCUS use by emergency physicians as well as CPOCUS course content and the American College of Emergency Physicians (ACEP) compendium [3,21]. These documents were reviewed in consultation with EM POCUS fellowship trained emergency physicians and a list of utilization questions was derived. The survey was pilot tested on 60 local emergency physicians and modified using an iterative feedback process.

The final survey comprised 24 open and closed-ended questions related to demographics, attitudes toward POCUS, POCUS utilization for CPOCUS core and advanced applications, and barriers to POCUS use. Respondents were asked to report on what percentage of patients and shifts they used POCUS. As well, respondents were asked to report on a five-point Likert scale how frequently they used POCUS for each application when applicable. Approval was obtained from both the Office of Research ethics at Western University as well as the Lawson Health Research Institute.

Data analysis

Survey data was collected via SurveyMonkey software (San Mateo, CA) and then subsequently transferred to Microsoft Excel 2016 for analysis (Microsoft Corp, Redmond WA). Standard descriptive statistics were calculated, and differences in mean POCUS usage between groups were measured using a one-way analysis of variance (ANOVA). Pearson correlations were conducted between demographic factors (years practicing medicine, number of shifts per week, and age of physician) and POCUS usage (reported as the percentage of shifts). 

## Results

Demographics

Responses were obtained from 20.8% (317/1520) of emergency physicians to whom the survey was sent. All provinces except Prince Edward Island were represented as shown in Table 1. Respondents were primarily male (66%) with a mean (standard deviation, SD) age of 42.5 (10.2). Respondents had been in independent practice for an average (SD) of 13.4 (10.5) years and worked an average (SD) of 11.9 (3.5) shifts per month.

**Table 1:** Respondent demographics based on province of practice, level of POCUS training, residency training type, hospital type, and community size Total number of respondents: 317 POCUS, point-of-care ultrasound; CPOCUS, Canadian point-of-care ultrasound society; FRCP, Fellow of the royal college of physicians of Canada; CCFP, certification in the college of family physicians; EM, emergency medicine; ANOVA, analysis of variance

Respondent Demographics		
Current Province	Percent	Count
Alberta	11.7%	37
British Columbia	13.9%	44
Manitoba	4.1%	13
New Brunswick	1.3%	4
Newfoundland and Labrador	2.2%	7
Nova Scotia	2.8%	9
Ontario	39.4%	125
Quebec	10.1%	32
Saskatchewan	2.8%	9
No Response/Other	11.7%	37
Current POCUS Training	Percent	Count
POCUS Fellowship	6.6%	21
CPOCUS Advanced	24.9%	79
CPOCUS Core	42.9%	136
Formal Training Only	22.7%	72
Informal Training Only	1.6%	5
No Response/Other	1.3%	4
Residency Training	Percent	Count
FRCPC (EM) Training	28.1%	89
CCFP-EM Training	44.8%	142
CCFP Training	10.4%	33
No Response/Other	16.7%	53
Current Hospital Type	Percent	Count
Academic Tertiary Care	51.1%	162
Non-Academic Tertiary Care	3.5%	11
Community (>30,000)	24.3%	77
Community (<30,000)	11.0%	35
No Response/Other	10.1%	32
Current Community Type	Percent	Count
Urban	62.1%	197
Suburban	8.2%	26
Small Town	11.1%	35
Rural & Remote	7.9%	25
No Response/Other	10.7%	34

Attitudes and training experience

Regarding when POCUS training occurred, 14.8% received training solely as a component of residency, 56.5% received training solely outside of residency, and 28.7% received training both as a component of residency and independently. Table 1 displays additional demographics such as the level of POCUS training, residency training, hospital type, and community type.

Current use and applications

POCUS was highly regarded by respondents with 80.4% believing it essential and the remainder believing it useful. Respondents reported using POCUS on an average (SD) of 68% (30%) of shifts and 23% (17%) of patients.

Table 2 shows the results of the ANOVAs. The level of POCUS training was significantly associated with higher reported POCUS usage (F = 15.84, df = 5, *p *< 0.001), while longer residency training was not (*p* > 0.05). Further, more advanced POCUS training offered at the centers, where respondents practiced was also significantly associated with higher reported POCUS usage (F = 2.83, df = 6, *P* < 0.05). Regarding the types of emergency medicine practice, neither hospital type nor community type was significantly associated with reported POCUS usage (*p* > 0.05). 

**Table 2:** Mean POCUS usage by percentage of shifts (column 2) and percentage of patients (column 4) according to emergency physician level of POCUS training, residency training type, type of POCUS training offered at the center of practice, academic training levels offered at center of practice, hospital type they practice in, and type of community they practice in Note: Indicates statistical significance *p < 0.05, **p < 0.01, ***p < 0.001 for one-way ANOVA POCUS, point-of-care ultrasound; CPOCUS, Canadian point-of-care ultrasound society; FRCP, Fellow of the royal college of physicians of Canada; CCFP, certification in the college of family physicians; EM, emergency medicine; ANOVA, analysis of variance

Variable	Usage Rate (% of Shifts)	F (df)	Usage Rate (% of Patients)	F (df)
POCUS Training		19.80 (5)***		15.84 (5)***
POCUS Fellowship	87.5%		42.9%	
CPOCUS Advanced (equivalent)	81.4%		28.5%	
CPOCUS Core (equivalent)	66.9%		20.2%	
Formal Training only	44.0%		13.7%	
Informal Training only	24.8%		6.4%	
Residency Training		1.04 (3)		1.91 (3)
FRCPC (EM)	68.7%		25.0%	
CCFP-EM	67.8%		21.6%	
CCFP	62.2%		16.8%	
Center POCUS Training Offered		3.65 (6)**		2.82 (6)*
POCUS Fellowship	70.7%		25.6%	
CPOCUS Advanced Certification (or equivalent)	75.8%		28.8%	
CPOCUS Core Certification (or equivalent)	68.8%		22.5%	
Formal training, no certification	40.0%		12.1%	
Informal training	69.6%		18.8%	
None	57.0%		18.1%	
Center Academic Training Offered		1.02 (6)		0.91 (6)
FRCP-EM Residency	67.6%		24.1%	
CCFP-EM Residency	72.1%		22.0%	
CCFP Residency	63.6%		17.9%	
Other Residencies	58.8%		21.7%	
Medical Students Only	53.3%		20.0%	
None	64.6%		23.8%	
Hospital Type		1.56 (4)		1.61 (4)
Academic Tertiary Care	69.0%		24.3%	
Non-Academic Tertiary Care	75.5%		23.4%	
Community (>30 000)	65.6%		19.8%	
Community (< 30 000)	59.1%		17.6%	
Community Type		1.18 (5)		0.48 (5)
Urban	68.3%		23.2%	
Suburban	65.1%		20.3%	
Small Town	69.3%		19.6%	
Rural	55.6%		20.8%	
Remote	60.4%		16.4%	

Correlation analyses were performed to assess differences in POCUS usage (as the percentage of shifts) by demographic factors. Number of shifts worked per month was positively associated with reported POCUS usage (r = 0.270, *p* < 0.001) while age was negatively associated with reported POCUS usage (r= -0.134, *p* < 0.05). Years practicing medicine was not significantly associated with reported POCUS usage (*p* > 0.05).

**Figure 1:**
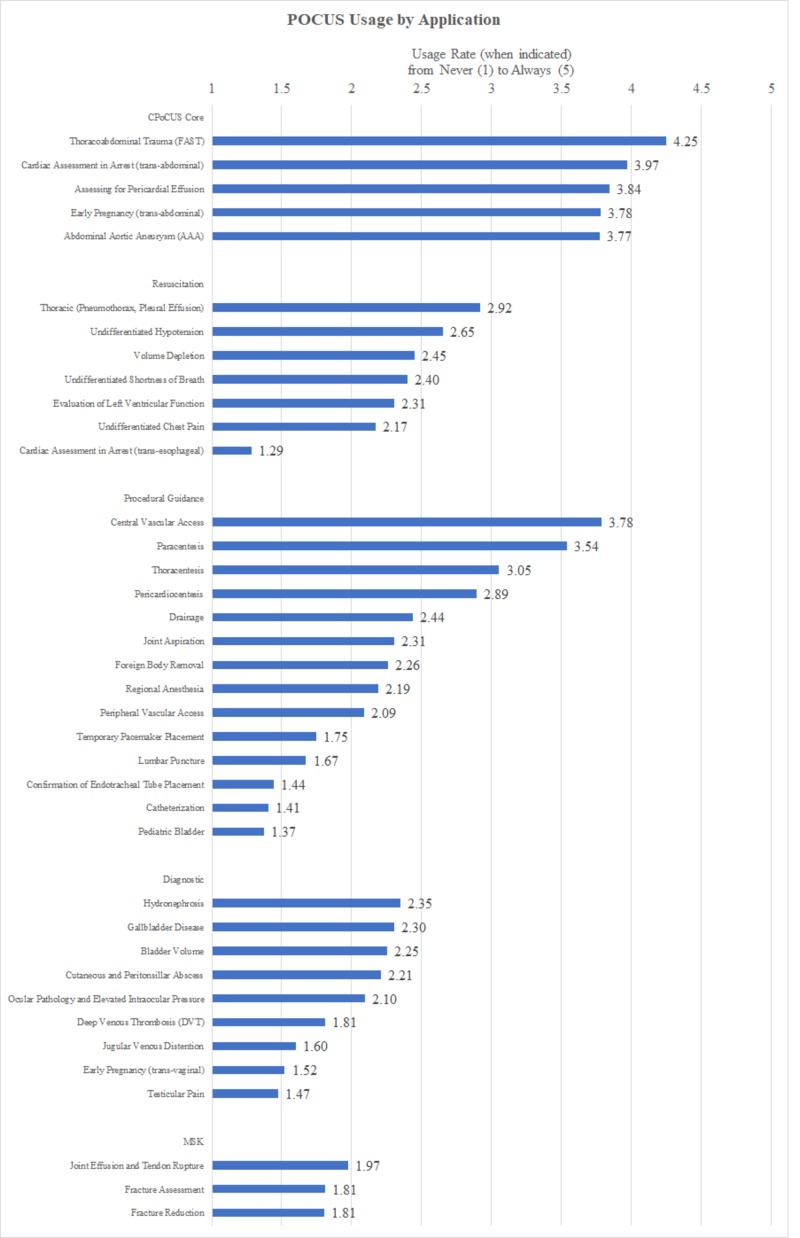
Rate of POCUS usage for various applications by emergency physicians Scale as follows: 1 = never (0%), 2 = rarely (<25%), 3 = regularly (25% to 75%), 4 = Usually (76% to 99%), 5 = Always (100%) MSK, musculoskeletal; POCUS, point-of-care ultrasound

Figure 1 outlines POCUS usage by application. POCUS was most widely adopted for core applications including FAST (4.25/5), assessment in cardiac arrest (trans-thoracic; 3.97/5), assessment for pericardial effusion (3.84/5), assessment in early pregnancy (trans-abdominal; 3.78/5), and AAA (3.77/5). The next most commonly used applications procedural guidance for central venous access (3.78/5) and paracentesis (3.54/5).

Barriers

The most commonly reported barriers to increased POCUS adoption were the lack of training (45.1%), lack of time/departmental flow requirements (19.2%), and lack of access to a POCUS machine (17.4%). Only 8.2% of respondents cited the lack of evidence as a barrier to further POCUS adoption. These reported barriers did not significantly vary based on either hospital or community type but, given the small numbers for each, were not analyzed for statistical significance.

## Discussion

Emergency physicians have been at the leading edge of POCUS adoption since its introduction in the 1980s. Results of this survey demonstrate that in Canadian EDs, POCUS is being used quite often. As seen in Figure 1, our respondents report usage of core applications of FAST, basic cardiac assessment, early pregnancy assessment, and AAA regularly to always. Further, given that, years in practice are negatively correlated with POCUS usage, including assessment in cardiac arrest and procedural guidance, POCUS usage rates are likely to continue increasing as physicians retire and are replaced by recent graduates who have never trained to complete these procedures and assessments “blind” without POCUS.

There is still tremendous potential for growth in POCUS adoption. As shown in Figure 1, many guideline-backed POCUS applications such as evaluation of acute dyspnea or fracture assessment have yet to become widely adopted [21-23]. Furthermore, additional applications continue to be developed, and thus, the opportunity for additional POCUS utilization is broader still. When taken with the finding that POCUS usage improves patient satisfaction and clinician confidence, increased POCUS adoption is a worthy goal.

Our study results indicate that increased POCUS training (both individual as well as the level of training offered at the center of practice) is associated with increased POCUS usage. Therefore, more widespread POCUS training, particularly in the less widely adopted advanced applications, could lead to higher POCUS utilization. Moreover, the lack of POCUS training was the most common barrier to increased POCUS utilization, which further supports this recommendation. Given that POCUS is a core competency in emergency medicine, core training should continue to be provided in residency; however, given the rapid pace of POCUS development, continuing professional development is also essential to remain up to date. Incorporation of POCUS into the competency-based curricula [20] should thus focus on the current applications while also emphasizing the need for continuing professional education as this modality continues to evolve.

Limitations

Small sample size due to a low response rate is a limitation, this may have caused our study to be under-powered. However, the response rate is consistent with those observed in other surveys of physicians [24-25]. It is also similar to previously reported CAEP response rates for similar studies [26-27]. It is also important to note that our survey was not formally validated and since it involved retrospective self-reporting some respondents may, in fact, respond differently than how they actually practice. In addition, a high proportion of our respondents practice in academic centers who may perceive greater value integrating POCUS into ED care and training. Self-selection bias may have resulted in respondents that have a higher interest in POCUS, who may perceive higher importance of POCUS overall. However, our respondents practice across a wide variety of emergency department settings and have a high variability of years of clinical experience. 

## Conclusions

POCUS is highly regarded by emergency physicians with most having formal POCUS training. This study demonstrates that POCUS training and utilization appear to have increased over the last decade and provide both a foundation for future POCUS research and direct future training programs.
